# Bayesian species delimitation reveals generalist and specialist parasitic wasps on *Galerucella* beetles (Chrysomelidae): sorting by herbivore or plant host

**DOI:** 10.1186/1471-2148-13-92

**Published:** 2013-04-27

**Authors:** Peter A Hambäck, Elisabet Weingartner, Lars Ericson, Lisa Fors, Anna Cassel-Lundhagen, Johan A Stenberg, Johannes Bergsten

**Affiliations:** 1 Department of Ecology, Environment and Plant Sciences, Stockholm University, Stockholm, SE-106 91, Sweden; 2 Department of Ecology and Environmental Science, Umeå University, Umeå, SE-901 87, Sweden; 3 Swedish University of Agricultural Sciences, Department of Ecology, Box 7044, Uppsala, SE-750 07, Sweden; 4 Department of Entomology, Swedish Museum of Natural History, Box 50007, Stockholm, SE-10405, Sweden

## Abstract

**Background:**

To understand the ecological and evolutionary consequences of species interactions in food webs necessitates that interactions are properly identified. Genetic analyses suggest that many supposedly generalist parasitoid species should rather be defined as multiple species with a more narrow diet, reducing the probability that such species may mediate indirect interactions such as apparent competition among hosts. Recent studies showed that the parasitoid *Asecodes lucens* mediate apparent competition between two hosts, *Galerucella tenella* and *G. calmariensis*, affecting both interaction strengths and evolutionary feedbacks. The same parasitoid was also recorded from other species in the genus *Galerucella*, suggesting that similar indirect effects may also occur for other species pairs.

**Methods:**

To explore the possibility of such interactions, we sequenced mitochondrial and nuclear genetic markers to resolve the phylogeny of both host and parasitoid and to test the number of parasitoid species involved. We thus collected 139 *Galerucella* larvae from 8 host plant species and sequenced 31 adult beetle and 108 parasitoid individuals.

**Results:**

The analysis of the *Galerucella* data, that also included sequences from previous studies, verified the five species previously documented as reciprocally monophyletic, but the Bayesian species delimitation for *A. lucens* suggested 3–4 cryptic taxa with a more specialised host use than previously suggested. The gene data analyzed under the multispecies coalescent model allowed us to reconstruct the species tree phylogeny for both host and parasitoid and we found a fully congruent coevolutionary pattern suggesting that parasitoid speciation followed upon host speciation.

**Conclusion:**

Using multilocus sequence data in a Bayesian species delimitation analysis we propose that hymenopteran parasitoids of the genus *Asecodes* that infest *Galerucella* larvae constitute at least three species with narrow diet breath. The evolution of parasitoid *Asecodes* and host *Galerucella* show a fully congruent coevolutionary pattern. This finding strengthens the hypothesis that the parasitoid in host search uses cues of the host rather than more general cues of both host and plant.

## Background

Consumer species feeding on multiple resources may indirectly mediate interactions among their resources [1-3], and thereby affect both trait evolution of interacting species and eventually speciation. A classic example is apparent competition, where one species affects the density of other species through an increased density of the shared consumer [4-9]. We are interested in the potential for such interactions among a set of chrysomelid beetles (*Galerucella* spp.) and their parasitoids, including the ecological and evolutionary consequences. Previous studies within this system show that parasitism rates may be very high, and that parasitoids may mediate apparent competition between hosts [10-12]. The literature suggests that the same parasitoid species *Asecodes lucens* (Nees) (Hymenoptera: Eulophidae) attacks all *Galerucella* species [10,11,13,14], and parasitoid mediated interaction could therefore involve additional species. However, cryptic species are common in parasitoid systems and diet breadth may be more narrow than literature information suggest [15-18]. For instance, Smith et al. [15] showed that 17 tachinid fly species that were thought to be broad generalist parasitoids on lepidopteran larvae consist of 32 species with a more narrow diet breadth. Similarly, *Apanteles leucostigmus*, thought to be a generalist parasitoid on at least 32 species of Hesperiidae caterpillars, revealed 36 distinct Barcode clusters interpreted as provisional specialist species by Smith et al. [19].

DNA-based species delimitation provides a novel method for discovering cryptic species where supposedly generalist consumers are shown to be morphologically more or less identical specialist consumers. However, the standard DNA barcoding protocols used to infer putative species are based on single loci and do not test species limits with models tracking speciation and population genetic processes e.g., [20]. An improved protocol such as the General Mixed Yule Coalescent model (hereafter GMYC) treats species-to-population transitions in a gene tree [21]. However, GMYC also assumes reciprocal monophyly of species and that the gene tree is known without error, ignoring that non-monophyletic species in gene trees are common [22,23] and that the time needed for complete lineage sorting of ancestral polymorphism is substantial ([24], see also [25,26]). When deriving the species tree, one approach is to concatenate multiple loci into a superlocus by assuming that gene trees are consistent among each other and with the species tree. An alternative approach is to explicitly model the coalescent process of each gene tree separately under the constraint of a common species tree [27-31]. This later approach is implemented in BPP, a Bayesian species delimitation method [32], that in a recent evaluation came out as the most accurate species delimitation method under varying conditions of divergence time, number of loci and migration [33]. BPP offers an exciting new tool to detect recent speciation events and cryptic species and is particularly suitable for host-parasitoid systems due to the prerequisite of an informed prior (a “guide tree”; Yang and Rannala 2010, see material and methods).

Cryptic species are thought to be more common in groups where chemical senses are more highly developed than vision, such as most insects, because changes in chemical communication do not necessitate morphological changes for the evolution of reproductive barriers between sibling species [34]. Cryptic speciation in parasitic hymenoptera is particularly interesting as this group is often reported to mediate indirect interactions between host species [5,35-37], but also because host finding in parasitoids involves both host volatiles and volatiles from the plant associated to the herbivore host [38]. Host finding is often described along a detectability-reliability axis, where plant volatiles associated to the host are more abundant than host volatiles, but also have a lower reliability. Parasitoids using a combination of host and plant volatiles may then face greater difficulties in finding hosts on different host plants compared to parasitoid species that only use host volatiles for host finding. As a consequence, generalist parasitoid species may have to use more general cues for host finding and this may, in turn, result in less efficient host search cf. [39]. Due to such trade-offs, specialisation and speciation cannot be separated from the chemical ecology underlying host finding.

The chrysomelid beetle genus *Galerucella* contains a mixture of monophagous and polyphagous herbivore species that occur commonly in wetlands across the northern hemisphere. Within this genus, in contrast to the generally accepted dogma for speciation in herbivore insects, host shifts have not necessarily involved closely related host plant species. Speciation in *Galerucella* has instead occurred between co-occurring wetland plants of such distant relations as Salicaceae, Primulaceae, Lythraceae, Rosaceae, Polygonaceae, Nymphaeaceae and Betulaceae. It is not likely that such distantly related plant species share much volatile profiles beyond green leaf volatiles [40], and even these are likely to occur in very different proportions (supported by unpublished chemical analyses in our research group). One may then ask how parasitoids face this chemical diversity and yet manage to find hosts and do host shifts. Most *Galerucella* species are only parasitized during the larval stage by *Asecodes* spp, but some species are also attacked by other hymenopteran taxa. The female parasitoids mainly attack the early larval stages, and lay multiple eggs in one larvae. In our study area, hosts and parasitoids typically have one generation per year but exceptions do occur.

In this paper, we use multilocus sequence data to test whether *A. lucens* attacking *Galerucella* larvae represent one generalist or multiple specialist species. For this purpose, we collected *Galerucella* larvae from most of their respective host plant species, reared them to adults to collect the parasitoids and sequenced them. For comparative purposes, we also sequenced three genes of the collected *Galerucella* species, to complement previous analyses [41]. Bayesian species delimitation suggested that *A. lucens* consists of 3–4 cryptic taxa with a more specialised host use than previously suggested. The gene data analyzed under the multispecies coalescent model allowed us to construct the species tree for both host and parasitoid and we found a fully congruent coevolutionary pattern suggesting that parasitoid speciation followed upon host speciation.

## Methods

### Study species and sampling

**Figure 1:**
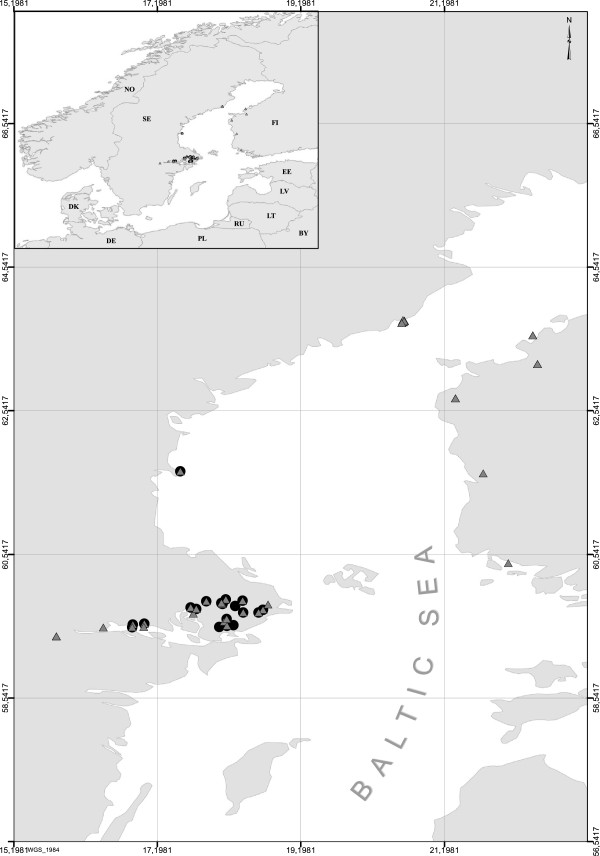
**Sampling locations for *Galerucella *and *Asecodes *specimens in Sweden and Finland.** Circles refer to sites were *Galerucella* were sampled and triangles refer to the collection sites of *Asecodes*.

Specimens of *Galerucella* (Coleoptera) and *Asecodes lucens* (Nees) (Hymenoptera) were collected in Sweden and Finland during 2011 (Figure 1, for details see Additional file 1: Table SI-1). Larvae of *Galerucella* were collected on their host plant, and subsequent rearings provided either adult beetle or parasitoid specimens in the lab. One species, *G. nymphaeae*, was collected but was not found to be parasitized and is therefore excluded from the data set. All specimens were stored in 99% ethanol. The sampling in Sweden was mainly from Uppland and Västmanland but parasitoids infesting *G. tenella* and *G. calmariensis* were also sampled from northern localities. In particular, a systematic sampling was performed in Skeppsvik archipelago in Umeå, where parasitism rates are typically much higher than in southern localities (> 70% vs. < 10%).

As outgroups to the *Galerucella* dataset we downloaded sequences of *Trirhabda bacharidis*, *Pyrrhalta* spp. and *Ophraella communa* (Galerucinae, Chrysomelidae) from GenBank. *Galerucella* sequences available on GenBank were also downloaded and included in our dataset (see Additional file 1: Table SI-1). In the *Asecodes* dataset we included part of the CO1 sequence of *Horismenus missouriensis, H. petiolatus* and *Pediobius* spp*.* (Entedoninae, Eulophidae) as well as *Quadrastichus haitiensis* (Tetrastichinae, Eulophidae) as outgroup species (downloaded from GenBank). Two *Pediobius* specimens were also reared from *G. sagittariae*, an host association that was not previously recorded (Additional file 1: Table SI-1).

### DNA extraction, amplification and sequencing

#### Extractions

Specimens for DNA extraction were chosen to represent samples from different collection sites and, in the case of *G. sagittariae*, to represent different host plant preferences. We used QIAamp® DNA Mini Kit (QiaGen) for the DNA extraction, where one to two legs per specimen were used for *Galerucella* while two to four legs were used for *Asecodes*. Legs were put in ATL lysis buffer and proteinase K and left in 56°C overnight. The purification procedure was carried out according to the manufacturer’s protocol except that the *Asecodes* extracts were eluted in 100 μl AE buffer.

#### DNA amplification

For both *Asecodes* and *Galerucella* we amplified the mitochondrial DNA of cytochrome oxidase 1 (CO1) gene and two nuclear genes; the internal transcribed spacer (ITS2) from the nuclear ribosomal region and the D2 region of the 28S ribosomal subunit. CO1 was amplified in two fragments without overlap using the primer pairs LCO-1490 & HCO-2198 [42] and C1-J-2183 (Jerry) & TL2-N-3014 (Pat) [43]. For the amplification of the nuclear genes we used the primer pairs ITS2f & ITS2r [44] and 28S_D2_F & 28S_D2_R [44] respectively. For *Asecodes* we also amplified the nuclear fragment of *phosphogluconate dehydrogenase* (PGD) using the primer pair PGD_hym_3F & PGD_hym_intRb (Malm unpubl). PCR reactions were performed with Ready-ToGo™ PCR beads (Amersham Biosciences) in a total reaction volume of 25 μl only adding 1 μl each of forward- and reverse primers (10 μM), 2 μl template and dH_2_O. The cycling profile started with a 5 min denaturation step at 95°C, followed by 38 cycles of 30 s at 95°C, annealing for 30 s at 49°C (ITS2), 50°C (CO1), 60°C (28S), extension for 1 min at 72°C, and a final extension step of 8 min at 72°C. For amplification of PGD, 40 cycles were run, 45 s was used in the denaturation step and annealing was performed for 40 s at 54°C in each cycle. The results were visualized on an agarose gel and stained with GelRed™ (Life Technologies). The PCR products were cleaned using Exonuclease I and FastAP (Fermentas) and the sequencing reactions were performed with BigDye™ Terminator ver. 3.1 Cycle Sequencing Kit (Applied Biosystems). The same primers as for the PCR reactions were used. The sequencing reactions were then cleaned with DyeEx 96 kit (QIAGEN) and run on an ABI Prism 3100 Genetic Analyzer (Applied Biosystems). Genetic fragments were sequenced in both forward and reverse directions. To align the ITS sequences we used the program Mafft [45] and the iterative refinement method which incorporates global pairwise alignment information. All other sequences were aligned and edited by eye in BioEdit v7.0.9 [46].

#### Phylogenetic analyses

*Galerucella* terminals were assigned to species based on which host plant they were sampled as well as morphological identification. The *Asecodes* terminals were assigned to which host they parasitized; e.g., *Asecodes*/*G. lineola* refers to an *Asecodes* specimen hatched from *G. lineola*.

We used MrModeltest2 v.2.3 [47] in conjunction with PAUP [48] to select the best model for the Bayesian analyses, although the substitution rate matrix was not selected a priori (see below). For CO1 the 1^st^, 2^nd^ and 3^rd^ codon positions were analyzed separately. Substitution model details for the *Galerucella* and *Asecodes* datasets are specified in Additional file 1: Table SI-2. The Bayesian phylogenetic analyses were performed with MrBayes 3.1 [49,50] at the online Bioportal server (University of Oslo, Norway). The *Galerucella* dataset consisted of 53 terminals representing eleven *Galerucella* species and two outgroup taxa for the CO1 + nuclear gene dataset and 19 terminals representing five *Galerucella* species and two outgroup genera for the nuclear gene dataset. The *Asecodes* dataset (CO1 + nuclear genes) included 107 taxa and six outgroup taxa. Each gene was analyzed separately (except the 28s for *Asecodes*) as well as combined, to evaluate the relative information content in each gene fragment. In the datasets including more than one partition (CO1) or including several genes, the substitution model was set to “mixed”, which implements reversible-jump MCMC across the entire space of 203 reversible 4 × 4 nucleotide substitution models [51]. All model parameters except branch lengths and topology were unlinked and relative rates between partions were allowed with a rate multiplier. We made two separate runs completing 10 million generations, each with four incrementally heated chains (*T*=0.2) and where sampling was done every 1000^th^ generation. The first 2 500 trees from each run were discarded as burn-in and the remaining samples pooled before calculating the majority-rule consensus. We checked that the separate analyses had converged using the average deviation of split frequencies diagnostic (< 0.02 in all runs), and the potential scale reduction factor (close to 1.00 for all parameters).

We used the multispecies coalescent model [30] as implemented in *BEAST (‘Starbeast’) to infer the species tree from multiple gene trees for both *Galerucella* and *Asecodes*. In the multispecies coalescent model all model parameters are unlinked across loci, including the topology parameter, which allows the gene trees to differ in topology while being constrained by one and the same species tree. The *Galerucella* dataset consisted of 18 terminals from five species and for one mitochondrial (CO1) and two nuclear loci (28S and ITS). Substitution models were set according to selected model for each loci by MrModeltest [47]. We applied a strict clock model on branch lengths and calibrated the CO1 partition to 0.0177 substitutions per site per million years after a recent CO1 clock rate estimate of another Polyphaga beetle family [52].

The *Asecodes* dataset consisted of 38 terminals from four species (as identified by the Bayesian species delimitation method, see below) and for one mitochondrial (CO1) and three nuclear loci (PGD, ITS and 28S). We applied a strict clock model on branch lengths and calibrated the species tree by applying a normal distribution prior (mean = 4.4, stdev = 0.7) on the root node according to the 95% HPD interval of the estimated host root node age. The purpose of this calibration was to see if, under the assumption of contemporary speciation at the root node, branching events after the root node predate or postdate respective branching event in the host tree. While the latter would be in agreement with (but does not ascertain) cospeciation, the former would falsify any such hypothesis. Note that the substitution rate calibration in the *Galerucella* analysis is applied to the CO1 gene tree whereas the root node calibration in the *Asecodes* analysis is applied to the species tree. The latter was enabled by hand editing of the xml file following McCormack et al. [53].

Both analyses were run four times independently for 100 million MCMC generations and sampled every 1000 generation. Tracer [54] was used to examine the convergence across runs and the ESS values of sampled parameters. After 10% of each run was discarded as a burn-in, the remaining samples from all runs were pooled and the maximum clade credibility species tree was calculated using the mean node heights.

### Species delimitation analyses

To test if the *Asecodes* parasitoid on *Galerucella* consists of one generalist species parasitizing multiple host species, or several specialists each attacking a single host, we used a Bayesian species delimitation method described by Yang and Rannala [32]. The method as applied in the software BPP uses reversible-jump MCMC to sample different species delimitation models and estimate the posterior probability of each model. BPP accommodates both uncertainty in gene tree estimations by the Bayesian framework, and incomplete lineage sorting of species via the coalescent process model (both in contrast to the GMYC method below). This statistical power increases both with the addition of loci and individuals per species [55]. BPP needs a guide-tree as input for the analysis and both the number of terminals in the guide-tree and its topology can influence the result [56]. BPP only estimates the posterior probability of all possible ways to collapse the nodes in the guide-tree into fewer species. It does not test any alternative groupings of individuals into species that cannot be derived from collapsing a node in the guide-tree, nor does it test to split the dataset into more species than the given terminals in the guide-tree. The dependency on a guide-tree effectively limits the search space and was a practical necessity introduced by Yang and Rannala [32]. As such, BPP is not suitable for blind biodiversity assessment, but explicit *a priori* species hypotheses need to exist to avoid artificial divisions like the smallest geographical sampling localities [56]. In this respect, parasitoids have the advantage of having natural and biologically relevant units, the host species, which in our analysis serve as delimiters of the maximal number of terminal units (five). We also tested guide-trees with four and three terminals, which only confirmed the relevant subset of results from the five terminal guide-tree analysis and will not be discussed further. The guide-tree topology was taken from the *BEAST analysis, all nodes of which were supported by a posterior probability of 1.0 and hence we did not need to test the effect on the species delimitation of any alternative plausible topology as guide-tree (compare with 56).

To calculate the likelihood of different species delimitations, a model with two types of parameters is needed (the number of parameters depend on the number of species in the model): *Θ* = 4*Neμ* and *τ*. *Θ* is a product of the effective population size and the mutation rate and *τ* is the root age measured in expected number of substitutions per site to the tips. Disregarding variations in the mutation rate, a relatively large value of *Θ* means a large effective population size and a relatively large value of *τ* specifies an ancient divergence. Each terminal species in the species delimitation model and each ancestral internode (ancestral species) have a *Θ* parameter whereas each split between species has a *τ* parameter. In a Bayesian framework both parameters need a prior which is specified by a gamma distribution in BPP. The gamma distribution is defined by two numbers, *α* and *β*, and the mean of the distribution is *α*/*β*. The mean should not be orders of magnitude away from the posterior of the parameter as estimated from the data, otherwise the prior can cause flawed results [55]. As a diffuse prior we used *α* = 1 in all analysis and tested various combinations of *β* for *Θ* and *τ* around the estimated posterior for the parameters. The estimated posterior estimates were *Θ* ≈ 0.05 and *τ* ≈ 0.02 (see Additional file 1: Table SI-3). All analyses were therefore run in four combinations of prior gamma distributions: 1: *Θ*: G(1,10), *τ*: G(1,10), 2: *Θ*: G(1,10), *τ*: G(1,100), 3: *Θ*: G(1,100), *τ*: G(1,10) and 4: *Θ*: G(1,100), *τ*: G(1,100), representing all combinations where the mean is between 0.1 and 0.01 and which includes the posterior estimates for both parameters. We also tested the effect of the number of loci on the power of the method. To do that we sequentially added loci in the order 1 locus (CO1), 2 loci (CO1+PGD), 3 loci (CO1+PGD+ITS), 4 loci (CO1+PGD+ITS+28S). It could be argued that the adjacent ITS and 28S gene segments should be analyzed as linked, but because of the complex pattern of recombination and concerted evolution in the tandemly arrayed rDNA loci [57], we prefer to analyze them as separate loci. If in doubt about the independence between the two loci, the result from the 3 loci analyses can be consulted. Each analysis of number of loci and prior combination was run 4 times independently to check the convergence of results and variation of estimated posterior probability for species delimitation models. Each analysis consisted of 50000 MCMC generations sampled every 5^th^ generation and discarding 10% as burn-in. The two different algorithms for the reversible-jump MCMC described by Yang and Rannala [32] and named “0” and “1” gave similar results and we therefore only report the result from using the algorithm 0.

The GMYC analysis was performed in R statistical package with the help of ape, gee, MASS, paran and splits packages. The GMYC method takes as input an ultrametric gene tree which we derived from calculating the maximum clade credibility tree with mean node heights from the sampled CO1 gene trees in a BEAST analysis using a strict molecular clock and a codon-partitioned model according to results from MrModeltest. The method calculates the likelihood of a mixed Yule-coalescence model applied with a single threshold across the tree at every node. At each threshold, deeper nodes are modeled as speciation events according to a Yule model whereas each group of younger nodes is modeled separately according to the coalescent process model. The maximum likelihood solution generally identifies the ‘kink’ or point of increased diversification rate in a lineage-through-time plot of trees with multiple individuals per species for multiple species. This node serves as a species delimitation point under the assumption of species monophyly and not permitting any speciation event to be younger than the deepest coalescence. The maximum likelihood of the GMYC model was tested with a likelihood ratio test against a null model treating the entire tree as a single coalescent (i.e. against a one-species model).

## Results

In total the *Galerucella* alignment consisted of 2766 basepairs whereas the alignment of *Asecodes* contained 3089 basepairs (see Additional file 1: Table SI-4 for details).

### Phylogenetic analysis

**Figure 2:**
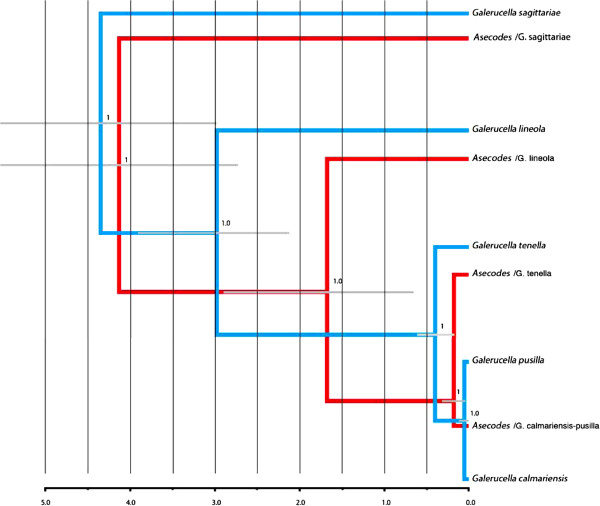
Topologies of *Galerucella *and *Asecodes *species, analysed with multispecies coalescent models in *BEAST. The analyses are based on sequences of CO1, 28S, ITS (*Galerucella* and *Asecodes*) and PGD (*Asecodes*). Branch labels indicate posterior probability values and the bars refer to the confidence interval of the node ages. The *Galerucella* dataset was rooted using a strict clock while the rooting of *Asecodes* was calibrated after the estimated age of *Galerucella*. The *Asecodes* specimens are coded as “*Asecodes* /the name of the *Galerucella* species that is parasitized”.

**Figure 3:**
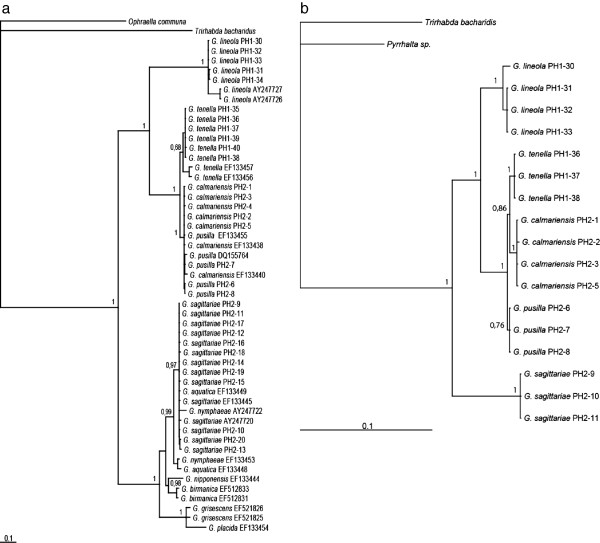
**Phylograms from Bayesian analysis of *Galerucella *species.** (**a**) Phylogram based on concatenated CO1, 28S and ITS sequences (2677 basepairs). (**b**) Phylogram based on the nuclear sequences of 28S and ITS (1203 basepairs). Branch labels indicate posterior probability values.

The multispecies coalescent model for *Galerucella* gave a well supported species tree with the topology (*G. sagittariae* (*G. lineola* (*G. tenella* (*G. pusilla* + *G. calmariensis*)))). Subgenus *Neogalerucella* (including *G. lineola*, *G. tenella*, *G. calmariensis* and *G. pusilla*) and subgenus *Galerucella* (including *G. sagittariae*) were monophyletic as defined by Borghuis *et al.* (2009), and *G. lineola* was basal in the *Neogalerucella* clade. All nodes had a posterior probability support of >0.99 and the age of the root node was estimated to 4.3 my (95% HPD: 3.0 – 5.9) with the mitochondrial clock calibration (Figure 2). The standard concatenated model with unconstrained branch lengths (non-clock model aka time-free model) of all genes combined resolved the species tree as the coalescent model to the same topology with high branch support (>0.99), but is here rooted using outgroups and includes a few additional species in the *G. sagittariae* group (Figure 3a). Analyzed alone, the mitochondrial CO1 gene tree is rooted differently compared to the nuclear (Figure 3b) and combined tree (CO1 and nuclear genes), which justifies the multispecies coalescent approach where gene trees are allowed to differ. It is interesting to note that the closely related *G. calmariensis* and *G. pusilla* were recovered as reciprocally monophyletic in the analyses of the nuclear genes but not by our CO1 data or by the mitochondrial data in Borghuis *et al.*[41]. The divergence date in the species tree is estimated to 77 000 years ago (95% HPD: 19000–148000).

The multispecies coalescent model for *Asecodes* with species defined according to the BPP analysis resolved the species tree to (*Asecodes*/*G. sagittariae* (*Asecodes*/*G. lineola* (*Asecodes*/*G. tenella* + *Asecodes*/*G. pusilla*/*calmariensis*)) with all posterior probability branch support 1.0 (Figure 2). Note that, as in the *Galerucella* analysis, the root was inferred from the clock model and not outgroups. As this is in perfect agreement with the phylogeny of the hosts, we calibrated the root node after the estimated age on the equivalent host chronogram node. For this we used the 95% highest posterior density of the host root node height to define a normal distribution (4.3, 0.7) as a prior on the parasite root node that takes the calibration uncertainty into account. This resulted in the node for the split of *Asecodes* on *G. lineola* and the node for the split of *Asecodes* on *G. tenella* to postdate the equivalent nodes in the host tree. The opposite would have been a potential falsifier of a cospeciation scenario.

**Figure 4:**
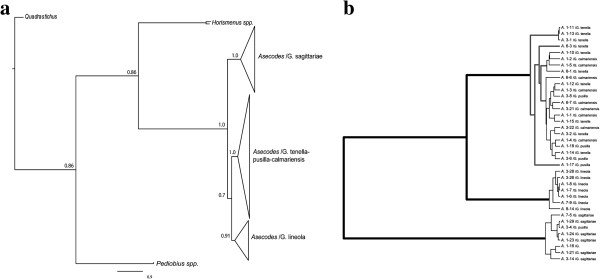
**Phylogram from Bayesian analysis of Asecode specimens.** (**a**) Phylogram based on concatenated CO1, PGD, 28S and ITS sequences (3124 basepairs). Due to short branches and lack of structure within the three clades, they are visualized as triangles codes as “*Asecodes* /the *Galerucella* species which are parasitized”. The three clusters include 31 (“*Asecodes*/*G. sagittariae*”), 57 “*Asecodes*/*G. tenella-pusilla-calmariensis*” and 19 “*Asecodes*/*G. lineola*” individuals. (**b**) Result of the GMYC analysis on an ultrametric CO1 gene tree from BEAST. Splits of thick black branches indicate speciation events whereas splits of thin black branches indicate within-species coalescence events. Grey branches are ambiguous in the GMYC analysis and all solutions from 3 to 7 species are included in a +/− 0.5 Log likelihood confidence interval. The single coalescence one-species model is rejected in a likelihood ratio test p=0.011.

With a standard concatenated model time-free (non-clock) model, and using outgroups to root the tree in a Bayesian analysis, three distinct clades were recovered corresponding to parasitoids on *G. sagittariae*, *G. lineola* and *G. tenella-calmariensis-pusilla*. The *Asecodes* specimens that infest *Galerucella sagittariae* were recovered as sister to the remaining *Asecodes* with high posterior probability, in agreement with the multispecies coalescent model. *Asecodes* on *G. sagittariae* and on *G. lineola* were each recovered as reciprocally monophyletic with high posterior probability support (1.0 and 0.91 respectively). *Asecodes* on *G. tenella, G. calmariensis and G. pusilla* however were not recovered as reciprocally monophyletic but were mixed in one large cluster, with strong branch support (1.0) (Figure 4). The CO1 gene tree was identical in topology to the combined tree whereas the nuclear dataset alone gave a completely unresolved topology in the ingroup because of few variable sites (Additional file 1: Figure SI-1). This gives an indication of the relative importance of different genes to the combined result. 28S had only a single variable site that separated *Asecodes* on *G. sagittariae* and *G. lineola* from the remaining parasitoids. Two variable positions defined the *G. sagittariae* parasitoids in the ITS gene fragment.

### Species delimitation

**Figure 5:**
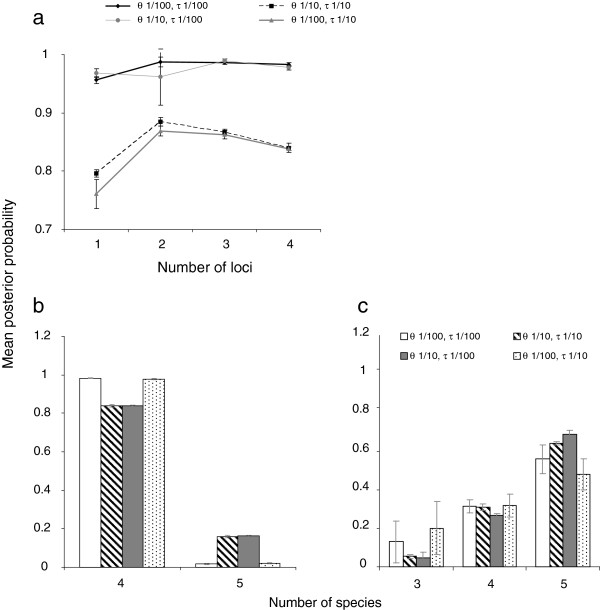
**Mean posterior probability value of species delimitation models.** The priors Θ and τ refer to the ancestral population size and age of root respectively. The bars indicate standard deviation. **a**. A four-species model and different number of loci for priors on Θ and τ. Loci: 1 - CO1; 2 - CO1, PGD, 3 - CO1, PGD, ITS; 4 - CO1, PGD, ITS, 28S. **b**. Analyses using all four genes resulted in highest support for the four-species model followed by the five-species model. **c**. Analyses using all four genes but only including the *Asecodes* specimens infesting *G. tenella* and *G. calmariensis* from the sympatric population in the northern part of Sweden (but including all other specimen infesting *G. sagittaria*, *G. lineola* and *G. pusilla*).

The BPP analyses yielded conclusive results and multiple runs with the same settings were in close agreement. Independent of the prior combinations used, the species delimitation model with highest posterior probability was the four-species model (Figure 5). The four-species model delimits a separate specialist *Asecodes* species each for *G. sagittariae*, *G. lineola* and *G. tenella* but a common species for *G. pusilla* and *G. calmariensis*. This model ranged in posterior probability from 0.73 to 0.99 (0.83 to 0.99 when analyzing all four genes) (Figure 5). The second best model was the five-species model where also *Asecodes* on *G. pusilla* and *G. calmariensis* was divided into separate species with a posterior probability of 0.002 to 0.27. The three-species model separating only *Asecodes* on *G. sagittariae*, *G. lineola* and remaining *Galerucella* had a marginal but non-zero support and the posterior probability for the two- and one-species model was 0 across all prior space investigated.

The effect of the number of loci was most noticeable between one (CO1) and two (CO1 + PGD) loci which yielded increased support for the four-species model. The third (ITS) and fourth (28S) loci contained only three and one variable sites respectively and had therefore marginal discriminatory power between species delimitation models. In fact, with the prior on Θ set to a relatively large population size G(1,10) the addition of ITS and 28S resulted in a decreased probability for the same model (Figure 5a).

The prior on the root age τ had very little effect on the posterior of delimitation models. With four loci and the prior combination Θ: G(1,10), τ: G(1,10) support for the best model was 0.83-0.84 and remained unchanged with Θ: G(1,10), τ: G(1,100). The effect of the prior on Θ was more noticeable and with the same priors on τ but with G(1,100) on Θ instead of G(1,10), the posterior probability increased to 0.97-0.98 for the best model. The support for three separate *Asecodes* species, on *G. sagittariae*, on *G. lineola* and on the remaining *Galerucella* species, is conclusive and never received a posterior probability of less than 1.0. The support for a fourth species *Asecodes* on *G. tenella* was strong but not entirely conclusive, as the three-species model treating *Asecodes* on *G. tenella, G. calmariensis* and *G. pusilla* as a single species also received some support (up to a posterior probability of 0.1 in one analysis). In addition, as the interpretation of allopatric populations with limited gene flow is a controversial issue we tested the BPP method on a sympatric subset of the data with only all *Asecodes* on *G. tenella* and *G. calmariensis* sampled from the same localities in northern Sweden. Used on sympatric populations, species defined unambiguously in the BPP analysis should be uncontroversial [55]. For the same four prior combinations, the posterior probability was more evenly spread across the three-, four- and five-species models with zero probability for the one- and two-species models (Figure 5c). The five-species model received mean posterior support of 0.41-0.72. That the dataset consist of at least three species, as opposed to one, is therefore conclusive, but to be conservative against oversplitting, the three-species model cannot be rejected even though it is the least supported of the three.

The GMYC model delimited five species with the threshold at the maximum likelihood solution (Figure 4b). The log likelihood of the GMYC model at the optimal threshold (247.2) was significantly better than the null model of a single coalescent (241.7) in a likelihood ratio test (p<0.011) and a one-species model could be rejected. However, the delimitation of three, four, five, six and seven species was less than 0.5 log likelihood units away from the maximum likelihood solution and should therefore be included within a reasonable confidence limit. In contrast to the BPP, the GMYC method does not accommodate incomplete lineage sorting, but assumes species monophyly. As the CO1 gene tree does not resolve *Asecodes* individuals parasitizing *G. tenella*, *G. calmariensis* or *G. pusilla* as reciprocally monophyletic, the delimitation of four or five species is not exactly the same as that assumed and tested in the BPP analysis. The GMYC method applied to the reduced sympatric dataset delimited three species at the maximum likelihood solution (data not shown).

## Discussion

The analysis of mitochondrial and nuclear genetic markers indicate that *Asecodes lucens* is not one generalist species but at least three species with a more narrow host use. Two parasitoid species seem to be specialist on their respective host, *Galerucella sagittariae* and *G. lineola*. The three remaining *Galerucella* species are seemingly attacked by the same parasitoid species, even though these parasitoids also show some population differentiation. There were tendencies that the parasitoids attacking *G. tenella*, which feeds on *F. ulmaria*, had diverged from the parasitoids attacking *G. calmariensis* and *G. pusilla*, which both feed on *L. salicaria*. The mitochondrial dating furthermore show that speciation in parasitoids are sequential events following speciation in their host, and a recent but perhaps not completed split between parasitoids attacking *G. tenella* and *G. calmariensis/pusilla*, would follow this pattern.

The derived phylogeny of *Galerucella* matches the previous analysis by Borghuis et al. [41] where the split between what is considered two subgenera, *Galerucella* and *Neogalerucella*, is most ancient. In our data set, the subgenus *Galerucella* only includes *G. sagittariae* whereas the other species belong to the subgenus *Neogalerucella*. Borghuis *et al.*[41] based their analysis only on mitochondrial gene fragments (CO1 and NADH-2), and found strong support for the monophyly of *G. tenella* and *G. lineola* but could not resolve *G. pusilla* and *G. calmariensis* as reciprocally monophyletic, indicating either a recent divergence or mitochondrial introgression*.* Our study also included two nuclear DNA fragments (the D2 region in 28S and ITS2), which provides a test of the alternative explanations. Although our nuclear DNA datasets were small, divergence between *G. pusilla* and *G. calmariensis* was strongly supported. These two taxa both use *Lythrum salicaria* (Lythraceae) as host plant, and both ecological and morphological information suggest that the two species are reproductively isolated. The male copulatory organs are distinctly different, the body size differs and both larval and adult colour differs [58,59]. In these characters, there is quite small overlap. In addition, Nokkala and Nokkala [60] found karyotypic differences which further support species status. In contrast to the conclusion drawn by Borghuis *et al.*[41], based on the results from mitochondrial gene fragments, the lack of monophyly in the mitochondrial genes for *G. pusilla* and *G. calmarensis* might rather indicate some recent “phenomenon” such as introgression, *Wolbachia* infestation etc. However, a larger sample from each population is necessary to establish if lineage sorting is indeed complete for the nuclear markers, which theoretically should sort slower than mitochondrial counterparts. Due to the maternal inheritage of the mitochondrion, effective population size is lower (1/4^th^ that of nuclear genes) and lineage sorting is therefore faster than for nuclear genes.

The Bayesian species delimitation analysis for the *A. lucens* group was performed on the same set of nuclear and mitochondrial genes as the analysis for *Galerucella*, with the addition of the nuclear gene PGD, and provided strong evidence for population differentiation. It is possible to conclude that *A. lucens* should be split at least into three species but additional data may strengthen the indication of further splits; the species delimitation analysis suggested 3–5 species. The molecular data have been confirmed by morphological studies that found differences in wing patterns among at least 3 taxa [61]. Comparisons with type specimens of available (synonymized) names suggested the identity of two taxa, *A. lucens* parasitizing *G. sagittariae* and *A. parviclava* (Thomson) parasitizing *G. tenella*, *G. calmariensis* and *G. pusilla*. A third species, parasitizing *G. lineola*, represents a new species named *A. lineophagum* Hansson & Hambäck [61]. There were no morphological characters supporting a further subdivision of *A. parviclava*.

The BPP method distinguishes populations as different species if the per generation migration rate *Nm* << 1 [55], and the interpretation is therefore unambiguous in sympatry but distance-decay patterns in allopatry may alone contribute to partial genetic isolation. Allopatry could explain why the analysis with all data included identified population differentiation between parasitoids collected on *G. tenella* versus *G. pusilla/calmariensis*. In the southern area, populations of *G. tenella* and *G. pusilla/calmariensis* are often found in slightly different habitats and were typically collected in different localities. In the northern area, where population differentiation was seemingly weaker, *G. tenella* and *G. calmariensis* co-occur on Baltic shore lines and were collected in the same localities. It is possible that the southern population of *A. parviclava* is in an early stage of speciation, but the current data are insufficient to confirm this suspicion.

The possibility of geographic variation in population differentiation of parasitoids is very interesting considering the previously documented parasitoid mediated indirect interactions between *G. tenella* and *G. calmariensis* in northern localities. Hambäck et al. [11] found that parasitism rates on *G. tenella* were higher at sites where this species co-occur with *G. calmariensis* compared with sites without *G. calmariensis*. The differences in parasitism rates translated into differences in the strength of interactions between the herbivore and its host plant [12]. In sites with *L. salicaria* and *G. calmariensis*, the attack rates on *F. ulmaria* by *G. tenella* were lower and the seed set were higher compared to sites without *L. salicaria*. Other studies show that this apparent competition between the *Galerucella* species may have evolutionary consequences for *F. ulmaria*. *Galerucella tenella* has a very strong impact on plant fitness and studies on differently aged populations suggest that *G. tenella* causes a shift in the population towards plants with a lower height and with higher concentrations of potential antiherbivore compounds [62]. In the field, the lower quality of *F. ulmaria* as food for the larvae of *G. tenella* caused the beetle to expand its diet towards other Rosaceae plants [63]. Preliminary data suggest that these evolutionary changes in *F. ulmaria* only occur on islands without *L. salicaria* (Ericson & Stenberg, unpublished data). The hypothesis for these effects was that the parasitoids use both *G. tenella* and *G. calmariensis*, even though the behavioural mechanisms are not fully understood [64]. It was therefore important to know whether parasitoids collected from the two host were indeed the same population and this seems to be the case. The potential for a larger population differentiation in southern localities suggest that a similar apparent competition is not likely in these localities, and the parasitism rates in these localities are also typically much lower (<10% vs >70%).

Speciation in the parasitoids for this system seem to follow the identity of the beetle host rather than the identity of the beetle’s host plant. This finding suggests that host finding or recognition cues originate from the larval host rather than from the host plant, such as beetle produced pheromones or beetle specific plant cues. Two contrasts in particular supports this view. First, *G. sagittariae* feed on multiple host plant species that are not closely related, but we nevertheless found no population differentiation among parasitoids hatching from larvae collected on different plants. Second, *G. sagittariae* and *G. tenella* are known to feed on the same host plant, but we find that their respective parasitoid belong to different taxa. It seems less likely that plant produced volatiles provide sufficient information for parasitoids to both differentiate between *G. sagittariae* and *G. tenella*, and at the same time to locate *G. sagittariae* on its different host plants. Beetle produced compounds therefore seem more likely. Previous studies show that at least two *Galerucella* species (*G. pusilla* and *G. calmariensis*) produce aggregation pheromones [65], but these compounds are produced by adult males and seem less likely to provide any information on the whereabouts of *Galerucella* larvae. To further understand the speciation process in *Asecodes* parasitoids, we are currently working to identify the compounds involved during the search process.

Speciation in parasitoids may follow different pathways according to published data, and the causes underlying this variability is not well understood. There are cases with host-association differentiation and seemingly tight cospeciation [66,67], similar to the one suggested in this study for the *Galerucella-Asecodes* system. In other cases, speciation in parasitoids is less well connected to the phylogeny of their host [68,69]. The current data both in this and most of the published cospeciation examples cannot differentiate whether this is ecological speciation where hosts or parasitoid species are not geographically isolated or cladogenesis where host and parasitoids are isolated in pairs [70]. Differences in the speciation pattern may arise because the parasitism process involves several steps that create barriers to parasitoids when their host switches diet. Diet switching and speciation in herbivorous insects is often connected to enemy free space where the host is more difficult to either find or exploit on an alternate plant [71-73]. Different plants may produce quite different volatile profiles upon damage and this may cause problems for parasitoid females to either find their host or even to identify the larvae as such. There are cases where a parasitoid species has geographic variability in the type of cues used during host search [74], but this area of research is poorly exploited. Besides affecting host search cues, plant quality is also known to affect herbivore immunocompetence and switching to an alternate plant may affect resistance to parasitoid attack. The *Galerucella-Asecodes* system seems ideal to study these processes in progress, both because host race formation is common and well documented among *Galerucella* beetles [75-77] and because interaction strengths between host and parasitoid show quite large geographic differences. Further studies on species interactions in this system however necessitates a better understanding also on the small scale population differentiation, to identify strengths of direct and indirect species interactions and their ecological and evolutionary consequences.

## Conclusion

Our analysis, using multilocus sequence data in a Bayesian species delimitation analysis, confirms the phylogenetic structure within the chrysomelid beetle genus *Galerucella* but also that the parasitoids attacking *Galerucella* larvae should be subdivided into at least three species. This subdivision has later been confirmed with morphological data. The previous literature suggested that one species of the genus *Asecodes* attacked *Galerucella* larvae but our analysis show that the parasitoids have a more narrow diet breadth. The evolution of parasitoid within *Asecodes* and the host *Galerucella* show a fully congruent coevolutionary pattern, even though the specific mechanism cannot be identified with the current data. The phylogeny of *Asecodes* show that host use is not connected to the plant species that host the beetle larvae as different host larvae on different plant species are attacked by different *Asecodes* species. This finding strengthens the hypothesis that the parasitoid uses cues of the host during search rather than more general cues of both host and plant. Previous studies within the *Asecodes-Galerucella* system has suggested that parasitoid-mediated interactions may be important for both beetle densities and plant performance. The observation that parasitoids in fact have a more narrow diet breadth suggests that such indirect interactions may be restricted to subsets of the food web involving these two genera.

### Availability of supporting data

The gene data set supporting the results of this article is available in Genbank (http://www.ncbi.nlm.nih.gov/genbank/), and accession numbers are found in Additional file 1: Table SI-1. The aligned matrices and trees of the *Galerucella* analyses (nuclear dataset and COI + nuclear dataset) and the *Asecodes* analysis (COI + nuclear dataset) are available at TREEBASE under URL http://purl.org/phylo/treebase/phylows/study/TB2:S14144.

## Competing interest

The authors declare that they have no competing interests.

## Authors’ contribution

PH designed the study, was involved in the field work and was responsible for the writing, EW carried out the molecular work and was involved in the statistical analysis and in writing the paper, LE, LS and JAS were involved in the field work and in the study design, ACL was involved in the design of the study and in the molecular work, JB was responsible for the statistical analysis and in the writing of the paper. All authors read and approved the final manuscript.

## Supplementary Material

Additional file 1: Table SI-1 Information about the specimens included in the analyses. **Table SI-2.** Substitution model parameters. **Table SI-3.** The effect of the prior on the posterior estimate of the theta and tau parameters. **Table SI-4.** Fragment lengths (bp), number of variable and parsimony informative sites and number of ingroup taxa for the *Galerucella* and *Asecodes* datasets. **Figure SI-1.** Relationship between *Asecodes* specimen based on sequences of the nuclear genes PGD, 28S and ITS with base pair changes of 28S and ITS. Click here for file
