# Supplementary material for: Bayesian species delimitation reveals generalist and specialist parasitic wasps on Galerucella beetles (Chrysomelidae): sorting by herbivore or plant host
Source: BMC Evol Biol. 2013 Apr 27;13:92. doi: 10.1186/1471-2148-13-92 (PMC3662573; doi:10.1186/1471-2148-13-92)
Supplement: Additional file 1: Table SI-1 — Information about the specimens included in the analyses. Table SI-2. Substitution model parameters. Table SI-3. The effect of the prior on the posterior estimate of the theta and tau parameters. Table SI-4. Fragment lengths (bp), number of variable and parsimony informative sites and number of ingroup taxa for the Galerucella and Asecodes datasets. Figure SI-1. Relationship between Asecodes specimen based on sequences of the nuclear genes PGD, 28S and ITS with base pair changes of 28S and ITS. [file 1471-2148-13-92-S1.doc]

**Table SI-1** Specimens included in the analyses. The analyses included four genes, CO1 = cytochrome oxidase 1 gene, 28S = the D2 region of the 28S ribosomal subunit, ITS = the internal transcribed spacer from the nuclear ribosomal region, PGD = the nuclear fragment of the *phosphogluconate dehydrogenase* gene. In addition, analysis of CO1 included two parts of the gene. Host plants include *Salix* spp, *Filipendula ulmaria* L., *Lythrum salicaria* L., *Comarum palustre* L., *Lysimachia thyrsiflora* L., *Polygonum* spp, *Trapa japonica* Flerow., *Persicaria hydropiper* (L.) Spach, *Persicaria amphibia* (L.) Gray, *Nuphar lutea* (L.) Sibth. & Sm., and *Rumex hydrolapathum* Huds..

| **Species** | **Id. no.** | **Collection site** | **Coordinates** | **Host plant** | **GenBank Accession no.** | | | |
| --- | --- | --- | --- | --- | --- | --- | --- | --- |
|  |  |  |  |  | **CO1** | **28S** | **ITS** | **PGD** |
| ***Galerucella*** |  |  |  |  |  |  |  |  |
| *G. lineola* | PH1-30 | Kärven,SE | 59.916°, 018.152° | *Salix* spp. | KC336451 | KC336420 |  |  |
| *G. lineola* | PH1-31 | Ludden, SE | 59.772°, 018.672° | *Salix* spp. | KC336452 | KC336421 | KC336436 |  |
| *G. lineola* | PH1-32 | Oppsjön, SE | 59.733°, 018.608° | *Salix* spp*.* | KC336454 | KC336422 | KC336437 |  |
| *G. lineola* | PH1-33 | Södersjön, SE | 59.852°, 018.107° | *Salix* spp*.* | KC336455 | KC336423 | KC336438 |  |
| *G. lineola* | PH1-34 | Kundbysjön, SE | 59.735°, 018.395° | *Salix* spp. | KC336453 |  |  |  |
| *G. tenella* | PH1-35 | Össeby, SE | 59.559°, 018.260° | *F. ulmaria* | KC336456 |  |  |  |
| *G. tenella* | PH1-36 | Sundängen, SE | 59.566°, 016.855° | *F. ulmaria* | KC336457 | KC336424 | KC336439 |  |
| *G. tenella* | PH1-37 | Sundängen, SE | 59.566°, 016.855° | *F. ulmaria* | KC336462 | KC336428 | KC336443 |  |
| *G. tenella* | PH1-38 | Svinnegarn, SE | 59.579°, 017.016° | *F. ulmaria* | KC336465 | KC336429 | KC433709 |  |
| *G. tenella* | PH1-39 | Enbyle, SE | 59.892°, 017.880° | *F. ulmaria* | KC336463 |  |  |  |
| *G. tenella* | PH1-40 | Liljekonvaljholmen, SE | 59.805°, 017.664° | *F. ulmaria* | KC336464 |  |  |  |
| *G. calmariensis* | PH2-1 | Norrfjärden, SE | 61.700°, 017.520° | *L. salicaria* | KC336458 | KC336425 | KC336440 |  |
| *G. calmariensis* | PH2-2 | Ängsö Dyvik, SE | 59.528°, 016.849° | *L. salicaria* | KC336461 | KC336427 | KC336442 |  |
| *G. calmariensis* | PH2-3 | Ludden, SE | 59.772°, 018.672° | *L. salicaria* | KC336459 | KC336426 | KC336441 |  |
| *G. calmariensis* | PH2-4 | Kärven, SE | 59.916°, 018.152° | *L. salicaria* | KC336460 |  |  |  |
| *G. calmariensis* | PH2-5 | Angarnsjön, SE | 59.550°, 018.169° | *L. salicaria* | KC336466 |  | KC336444 |  |
| *G. pusilla* | PH2-6 | Kärven, SE | 59.916°, 018.152° | *L. salicaria* | KC336467 | KC336430 | KC336445 |  |
| *G. pusilla* | PH2-7 | Kärven, SE | 59.916°, 018.152° | *L. salicaria* | KC336469 | KC336432 | KC336447 |  |
| *G. pusilla* | PH2-8 | Mörtsjön, SE | 59.644°, 018.166° | *L. salicaria* | KC336468 | KC336431 | KC336446 |  |
| *G. sagittariae* | PH2-9 | Fladen, SE | 59.866°, 018.090° | *L. vulgaris* | KC336470 | KC336433 | KC336448 |  |
| *G. sagittariae* | PH2-10 | Vallentunasjön, SE | 59.532°, 018.063° | *L. vulgaris* | KC336471 | KC336434 | KC336449 |  |
| *G. sagittariae* | PH2-11 | Sottern, SE | 59.897°, 018.387° | *L. vulgaris* | KC336472 | KC336435 | KC336450 |  |
| *G. sagittariae* | PH2-12 | Ålderskogen, SE | 59.826°, 018.284° | *L. vulgaris* | KC336474 |  |  |  |
| *G. sagittariae* | PH2-13 | Fläktan, SE | 59.782°, 017.741° | *C. palustre* | KC336479 |  |  |  |
| *G. sagittariae* | PH2-14 | Ludden, SE | 59.772°, 018.672° | *C. palustre* | KC336478 |  |  |  |
| *G. sagittariae* | PH2-15 | Angarnsjön, SE | 59.550°, 018.169° | *C. palustre* | KC336481 |  |  |  |
| *G. sagittariae* | PH2-16 | Kärven, SE | 59.916°, 018.152° | *C. palustre* | KC336475 |  |  |  |
| *G. sagittariae* | PH2-17 | Svinnegarn, SE | 59.579°, 017.016° | *L. thyrsiflora* | KC336473 |  |  |  |
| *G. sagittariae* | PH2-18 | Angarnsjön, SE | 59.550°, 018.169° | *L. thyrsiflora* | KC336476 |  |  |  |
| *G. sagittariae* | PH2-19 | Angarnsjön, SE | 59.550°, 018.169° | *Polygonum* | KC336480 |  |  |  |
| *G. sagittariae* | PH2-20 | Södersjön, SE | 59.852°, 018.107° | *Polygonum* | KC336477 |  |  |  |
| *G. lineola* |  |  |  |  | AY247727 |  |  |  |
| *G. lineola* |  |  |  |  | AY247726 |  |  |  |
| *G. tenella* |  | Zoerselbos, Belgium | | *F. ulmaria* | EF133457 |  |  |  |
| *G. tenella* |  | Tvärminne, FI |  | *F. ulmaria* | EF133456 |  |  |  |
| *G. pusilla* |  |  |  |  | DQ155764 |  |  |  |
| *G. calmariensis* |  | Tvärminne, FI |  | *L. salicaria* | EF133440 |  |  |  |
| *G. pusilla* |  | Roelofsven, The Netherlands | | *L. salicaria* | EF133455 |  |  |  |
| *G. calmariensis* |  | SE |  | *L. salicaria* | EF133438 |  |  |  |
| *G. nymphaeae* |  |  |  |  | AY247722 |  |  |  |
| *G. sagittariae* |  |  |  |  | AY247720 |  |  |  |
| *G. birmanica* |  |  |  |  | EF512833 |  |  |  |
| *G. birmanica* |  |  |  |  | EF512831 |  |  |  |
| *G. nipponensis* |  | Ohklahoma, Japan |  | *T. japonica* | EF133444 |  |  |  |
| *G. grisescens* |  |  |  |  | EF521826 |  |  |  |
| *G. grisescens* |  |  |  |  | EF521825 |  |  |  |
| *G. placida* |  | Warrel, Austria |  | *P. hydropiper* | EF133454 |  |  |  |
| *G. aquatica/sagittariae* | | Hjälstasjön, SE |  | *P. amphibium* | EF133449 |  |  |  |
| *G. sagittariae* |  | Alsjön, SE |  | *C. palustre* | EF133445 |  |  |  |
| *G. nymphaeae* |  | Trebelice, Czech |  | *N. lutea* | EF133453 |  |  |  |
| *G. aquatica* |  | Connemara, Ireland | | *R. hydrolapathum* | EF133448 |  |  |  |
|  |  |  |  |  |  |  |  |  |
| **Outgroups** |  |  |  |  |  |  |  |  |
| *Trirhabda bacharidis* | |  |  |  | AY242503 | AY243769 | AY514328 |  |
| *Ophraella communa* | |  |  |  | AB052082 |  |  |  |
| *Ophraella communa* | |  |  |  | JN208175 |  |  |  |
| *Pyrrhalta sp.* |  |  |  |  |  | AY243782 |  |  |
|  |  |  |  |  |  |  |  |  |
| ***Asecodes*** |  |  |  |  |  |  |  |  |
| *A. parviclava* | PH1-1 | Ludden | 59.772°, 018.672° | *G. calmariensis* | KC808603 | KC733719 | KC751454 |  |
| *A. parviclava* | PH1-2 | Kuggören | 61.699°, 017.520° | *G. calmariensis* | KC808604 | KC733720 | KC751455 | KC763897 |
| *A. parviclava* | PH1-3 | Norrfjärden | 61.699°, 017.520° | *G. calmariensis* | KC808605 | KC733721 | KC751456 |  |
| *A. parviclava* | PH1-4 | Kärven | 59.916°, 018.152° | *G. calmariensis* | KC808606 |  | KC751457 |  |
| *A. parviclava* | PH1-5 | Ängsö Dyvik | 59.528°, 016.849° | *G. calmariensis* | KC808607 |  | KC751458 |  |
| *A. parviclava* | PH3-21 | Kärven | 59.916°, 018.152° | *G. calmarensis* | KC808656 | KC733748 | KC751480 |  |
| *A. parviclava* | PH3-22 | Ängsö Dyvik | 59.528°, 016.849° | *G. calmarensis* | KC808657 | KC733749 | KC751481 |  |
| *A. parviclava* | PH3-23 | Ludden | 59.772°, 018.672° | *G. calmarensis* | KC808658 |  | KC751482 |  |
| *A. parviclava* | PH3-24 | Kuggören | 61.699°, 017.520° | *G. calmarensis* | KC808659 |  | KC751483 |  |
| *A. parviclava* | PH3-25 | Ängsö Dyvik | 59.528°, 016.849° | *G. calmarensis* | KC808660 |  | KC751484 |  |
| *A. parviclava* | PH6-5 | Skeppsvik, Umeå | 63.778°, 020.623° | *G. calmarensis* | KC808667 | KC733753 |  |  |
| *A. parviclava* | PH6-6 | Skeppsvik, Umeå | 63.779°, 020.612° | *G. calmarensis* | KC808668 | KC733754 |  | KC763917 |
| *A. parviclava* | PH6-7 | Skeppsvik, Umeå | 63.798°, 020.640° | *G. calmarensis* | KC808669 | KC733755 |  | KC763918 |
| *A. parviclava* | PH6-8 | Skeppsvik, Umeå | 63.787°, 020.628° | *G. calmarensis* | KC808670 | KC733756 |  |  |
| *A. parviclava* | PH6-16 | Skeppsvik, Umeå | 63.790°, 020.637° | *G. calmarensis* | KC808676 |  |  |  |
| *A. parviclava* | PH6-18 | Skeppsvik, Umeå | 63.768°, 020.604° | *G. calmarensis* | KC808677 |  |  |  |
| *A. parviclava* | PH1-10 | Liljekonvaljholmen | 59.805°, 017.664° | *G. tenella* | KC808612 |  |  | KC763899 |
| *A. parviclava* | PH1-11 | Enbyle | 59.892°, 017.880° | *G. tenella* | KC808613 |  | KC751460 | KC763900 |
| *A. parviclava* | PH1-12 | Asköviken | 59.524°, 016.445° | *G. tenella* | KC808614 |  | KC751461 | KC763901 |
| *A. parviclava* | PH1-13 | Enbyle | 59.892°, 017.880° | *G. tenella* | KC808615 | KC733725 | KC751462 |  |
| *A. parviclava* | PH1-14 | Svinnegarn | 59.579°, 017.016° | *G. tenella* | KC808616 | KC733726 | KC751463 | KC763902 |
| *A. parviclava* | PH1-15 | Liljekonvaljholmen | 59.805°, 017.664° | *G. tenella* | KC808617 | KC733727 | KC751464 |  |
| *A. parviclava* | PH3-1 | Enbyle | 59.892°, 017.880° | *G. tenella* | KC808636 | KC733738 | KC751472 |  |
| *A. parviclava* | PH3-2 | Svinnegarn | 59.579°, 017.016° | *G. tenella* | KC808637 | KC733739 | KC751473 |  |
| *A. parviclava* | PH3-3 | Asköviken | 59.524°, 016.445° | *G. tenella* | KC808638 |  | KC751474 |  |
| *A. parviclava* | PH6-1 | Skeppsvik, Umeå | 63.768°, 020.604° | *G. tenella* | KC808663 | KC733751 |  | KC763915 |
| *A. parviclava* | PH6-2 | Skeppsvik, Umeå | 63.787°, 020.628° | *G. tenella* | KC808664 |  |  |  |
| *A. parviclava* | PH6-3 | Skeppsvik, Umeå | 63.790°, 020.637° | *G. tenella* | KC808665 | KC733752 |  | KC763916 |
| *A. parviclava* | PH6-4 | Skeppsvik, Umeå | 63.772°, 020.624° | *G. tenella* | KC808666 |  |  |  |
| *A. parviclava* | PH6-9 | Skeppsvik, Umeå | 63.787°, 020.610° | *G. tenella* | KC808671 | KC733757 |  |  |
| *A. parviclava* | PH6-10 | Skeppsvik, Umeå | 63.792°, 020.646° | *G. tenella* | KC808672 |  |  |  |
| *A. parviclava* | PH6-11 | Skeppsvik, Umeå | 63.778°, 020.623° | *G. tenella* | KC808673 | KC733758 |  |  |
| *A. parviclava* | PH6-14 | Skeppsvik, Umeå | 63.779°, 020.612° | *G. tenella* | KC808674 |  |  | KC763919 |
| *A. parviclava* | PH6-15 | Skeppsvik, Umeå | 63.805°, 020.620° | *G. tenella* | KC808675 |  |  | KC763920 |
| *A. parviclava* | PH8-2 | Sandören, Finland | 63.594°, 022.432° | *G. tenella* | KC808698 |  |  | KC763927 |
| *A. parviclava* | PH8-3 | Hjulsta | 59.534°, 017.008° | *G. tenella* | KC808699 |  |  |  |
| *A. parviclava* | PH8-4 | Runsala, Finland | 60.423°, 022.090° | *G. tenella* | KC808700 |  |  |  |
| *A. parviclava* | PH8-5 | Älholmen | 59.402°, 015.792° | *G. tenella* | KC808701 |  |  |  |
| *A. parviclava* | PH8-6 | Onnenkoski, Finland | 61.674°, 021.740° | *G. tenella* | KC808702 |  |  |  |
| *A. parviclava* | PH8-7 | Röukas, Finland | 63.193°, 022.495° | *G. tenella* | KC808703 |  |  |  |
| *A. parviclava* | PH1-17 | Mörtsjön | 59.644°, 018.166° | *G. pusilla* | KC808619 | KC733728 | KC751466 |  |
| *A. parviclava* | PH1-18 | Mörbysjön | 59,605°, 018,127° | *G. pusilla* | KC808620 | KC733729 | KC751467 | KC763903 |
| *A. parviclava* | PH1-19 | Fläktan | 59.782°, 017.741° | *G. pusilla* | KC808621 | KC733730 | KC751468 |  |
| *A. parviclava* | PH1-20 | Mörbysjön | 59,605°, 018,127° | *G. pusilla* | KC808622 |  |  |  |
| *A. parviclava* | PH3-5 | Fläktan | 59.782°, 017.741° | *G. pusilla* | KC808640 |  | KC751476 |  |
| *A. parviclava* | PH3-6 | Mörtsjön | 59.644°, 018.166° | *G. pusilla* | KC808641 | KC733741 | KC751477 |  |
| *A. parviclava* | PH3-7 | Mörbysjön | 59,605°, 018,127° | *G. pusilla* | KC808642 |  |  | KC763910 |
| *A. parviclava* | PH3-8 | Mörbysjön | 59,605°, 018,127° | *G. pusilla* | KC808643 | KC733742 | KC751478 |  |
| *A. parviclava* | PH7-17 | Röukasträsk, Finland | 63.193°, 022.495° |  | KC808689 |  |  | KC763924 |
| *A. parviclava* | PH7-18 | Hinjärv, Finland | 62.716°, 021.351° |  | KC808690 |  |  |  |
| *A. parviclava* | PH7-19 | Hinjärv, Finland | 62.716°, 021.351° |  | KC808691 |  |  | KC763925 |
| *A. parviclava* | PH7-20 | Röukasträsk, Finland | 63.193°, 022.495° |  | KC808692 |  |  |  |
| *A. parviclava* | PH7-21 | Röukasträsk, Finland | 63.193°, 022.495° |  | KC808693 |  |  |  |
| *A. parviclava* | PH7-22 | Röukasträsk, Finland | 63.193°, 022.495° |  | KC808694 |  |  |  |
| *A. parviclava* | PH7-23 | Röukasträsk, Finland | 63.193°, 022.495° |  | KC808695 |  |  |  |
| *A. parviclava* | PH7-24 | Röukasträsk, Finland | 63.193°, 022.495° |  | KC808696 |  |  |  |
| *A. parviclava* | PH7-25 | Röukasträsk, Finland | 63.193°, 022.495° |  | KC808697 |  |  | KC763926 |
| *A. lineophagum* | PH1-6 | Kundbysjön | 59.735°, 018.395° | *G. lineola* | KC808608 | KC733722 |  |  |
| *A. lineophagum* | PH1-7 | Ludden | 59.772°, 018.672° | *G. lineola* | KC808609 | KC733723 |  | KC763898 |
| *A. lineophagum* | PH1-8 | Sundängen | 59.566°, 016.855 | *G. lineola* | KC808610 | KC733724 | KC751459 |  |
| *A. lineophagum* | PH1-9 | Mörtsjön | 59.644°, 018.166° | *G. lineola* | KC808611 |  |  |  |
| *A. lineophagum* | PH3-26 | Liljekonvaljholmen | 59.805°, 017.664° | *G. lineola* | KC808661 | KC733750 | KC751485 | KC763914 |
| *A. lineophagum* | PH3-27 | Haknäs | 59.718°, 017.699° | *G. lineola* | KC808662 |  |  |  |
| *A. lineophagum* | PH3-28 | Ludden | 59.772°, 018.672° | *G. lineola* | KC808632 | KC733737 |  | KC763909 |
| *A. lineophagum* | PH3-29 | Kärven | 59.916°, 018.152° | *G. lineola* | KC808633 |  |  |  |
| *A. lineophagum* | PH3-31 | Sundängen | 59.566°, 016.855 | *G. lineola* | KC808634 |  |  |  |
| *A. lineophagum* | PH3-32 | Ängsö Dyvik | 59.528°, 016.849° | *G. lineola* | KC808635 |  |  |  |
| *A. lineophagum* | PH7-8 | Hinjärv, Finland | 62.716°, 021.351° | *G. lineola* | KC808680 |  | KC751486 |  |
| *A. lineophagum* | PH7-9 | Hinjärv, Finland | 62.716°, 021.351° | *G. lineola* | KC808681 |  | KC751487 | KC763922 |
| *A. lineophagum* | PH7-10 | Älholmen | 59.402°, 015.792° | *G. lineola* | KC808682 |  |  |  |
| *A. lineophagum* | PH8-9 | Hinjärv, Finland | 62.716°, 021.351° | *G. lineola* | KC808704 |  |  | KC763928 |
| *A. lineophagum* | PH8-10 | Älholmen | 59.402°, 015.792° | *G. lineola* | KC808705 |  |  |  |
| *A. lineophagum* | PH8-11 | Sandören, Finland | 63.594°, 022.432° | *G. lineola* | KC808706 |  |  |  |
| *A. lineophagum* | PH8-12 | Sandören, Finland | 63.594°, 022.432° | *G. lineola* | KC808707 |  |  |  |
| *A. lineophagum* | PH8-13 | Sandören, Finland | 63.594°, 022.432° | *G. lineola* | KC808708 |  |  |  |
| *A. lineophagum* | PH8-14 | Sandören, Finland | 63.594°, 022.432° | *G. lineola* | KC808709 |  | KC751490 | KC763929 |
| *A. lucens* | PH1-16 | Svinnegarn | 59.579°, 017.016° |  | KC808618 |  | KC751465 |  |
| *A. lucens* | PH1-21 | Mörtsjön | 59.644°, 018.166° | *G. sagittariae* | KC808623 | KC733731 |  | KC763904 |
| *A. lucens* | PH1-23 | Sottern | 59.897°, 018.387° | *G. sagittariae* | KC808625 | KC733733 |  | KC763906 |
| *A. lucens* | PH1-24 | Fläktan | 59.782°, 017.741° | *G. sagittariae* | KC808626 | KC733734 |  |  |
| *A. lucens* | PH1-25 | Kärven | 59.916°, 018.152° | *G. sagittariae* | KC808627 |  |  |  |
| *A. lucens* | PH1-26 | Fläktan | 59.782°, 017.741° | *G. sagittariae* | KC808628 |  |  |  |
| *A. lucens* | PH1-27 | Ludden | 59.772°, 018.672° | *G. sagittariae* | KC808629 |  |  |  |
| *A. lucens* | PH1-29 | Södersjön | 59.852°, 018.107° | *G. sagittariae* | KC808631 | KC733736 | KC751471 | KC763908 |
| *A. lucens* | PH3-4 | Svinnegarn | 59.579°, 017.016° |  | KC808639 | KC733740 | KC751475 |  |
| *A. lucens* | PH3-9 | Mörbysjön | 59,605°, 018,127° | *G. sagittariae* | KC808644 |  |  |  |
| *A. lucens* | PH3-10 | Mörtsjön | 59.644°, 018.166° | *G. sagittariae* | KC808645 |  |  |  |
| *A. lucens* | PH3-11 | Kärven | 59.916°, 018.152° | *G. sagittariae* | KC808646 |  |  |  |
| *A. lucens* | PH3-12 | Brosjön | 59.847°, 018.742° | *G. sagittariae* | KC808647 | KC733743 |  |  |
| *A. lucens* | PH3-13 | Södersjön | 59.852°, 018.107° | *G. sagittariae* | KC808648 | KC733744 |  |  |
| *A. lucens* | PH3-14 | Angarn | 59.550°, 018.169° | *G. sagittariae* | KC808649 | KC733745 | KC751479 | KC763911 |
| *A. lucens* | PH3-15 | Svinnegarn | 59.579°, 017.016° | *G. sagittariae* | KC808650 | KC733746 |  |  |
| *A. lucens* | PH3-16 | Fladen | 59.866°, 018.090° | *G. sagittariae* | KC808651 | KC733747 |  |  |
| *A. lucens* | PH3-17 | Ludden | 59.772°, 018.672° | *G. sagittariae* | KC808652 |  |  |  |
| *A. lucens* | PH3-18 | Sottern | 59.897°, 018.387° | *G. sagittariae* | KC808653 |  |  | KC763912 |
| *A. lucens* | PH3-19 | Oppsjön | 59.733°, 018.608° | *G. sagittariae* | KC808654 |  |  |  |
| *A. lucens* | PH3-20 | Fläktan | 59.782°, 017.741° | *G. sagittariae* | KC808655 |  |  | KC763913 |
| *A. lucens* | PH7-5 | Älholmen | 59.402°, 015.792° | *G. sagittariae* | KC808678 | KC733759 |  | KC763921 |
| *A. lucens* | PH7-6 | Hinjärv, Finland | 62.716°, 021.351° | *G. sagittariae* | KC808679 | KC733760 |  |  |
| *A. lucens* | PH7-11 | Sandören, Finland | 63.594°, 022.432° | *G. sagittariae* | KC808683 |  |  |  |
| *A. lucens* | PH7-12 | Sandören, Finland | 63.594°, 022.432° | *G. sagittariae* | KC808684 |  |  |  |
| *A. lucens* | PH7-13 | Älholmen | 59.402°, 015.792° | *G. sagittariae* | KC808685 |  | KC751488 | KC763923 |
| *A. lucens* | PH7-14 | Sandören, Finland | 63.594°, 022.432° | *G. sagittariae* | KC808686 |  |  |  |
| *A. lucens* | PH7-15 | Sandören, Finland | 63.594°, 022.432° | *G. sagittariae* | KC808687 |  |  |  |
| *A. lucens* | PH7-16 | Sandören, Finland | 63.594°, 022.432° | *G. sagittariae* | KC808688 |  | KC751489 |  |
| *A. lucens* | PH8-15 | Älholmen | 59.402°, 015.792° | *G. sagittariae* | KC808710 |  |  | KC763930 |
| *A. lucens* | PH8-16 | Sandören, Finland | 63.594°, 022.432° | *G. sagittariae* | KC808711 |  |  | KC763931 |
|  |  |  |  |  |  |  |  |  |
| **Outgroup** |  |  |  |  |  |  |  |  |
| *Horismenus missouriensis* | |  |  |  | EU435168 |  |  |  |
| *Horismenus petiolatus* | |  |  |  | HM365039 |  |  |  |
| *Quadrastichus haitiensis* | |  |  |  | FJ872115 |  |  |  |
| *Pediobius sp.* |  |  |  |  | HQ107668 |  |  |  |
| *Pediobius sp.* | PH1-22 | Vallentunasjön, SE | 59.532°, 018.063° | *G. sagittariae* | KC808624 | KC733732 | KC751469 | KC763905 |
| *Pediobius sp.* | PH1_28 | Mörtsjön, SE | 59.644°, 018.166° | *G. sagittariae* | KC808630 | KC733735 | KC751470 | KC763907 |

**Table SI-2.** Substitution model parameters estimated from MrModeltest (Nylander, 2004). The models of the first and second parts of CO1 (*Asecodes*) were estimated separately and thus the models of the 3^rd^ position refer to the first part and second part respectively.

|  | Model | Rates | State frequency parameters |
| --- | --- | --- | --- |
| ***Galerucella*** |  |  |  |
| CO1, 1^st^ | GTR | Equal | Dirichlet (1,1,1,1) |
| CO1, 2^nd^ | F81 | Equal | Dirichlet (1,1,1,1) |
| CO1, 3^rd^ | GTR | Gamma | Dirichlet (1,1,1,1) |
| 28S | GTR | Equal | Fixed (equal) |
| ITS | GTR | Gamma | Dirichlet (1,1,1,1) |
| ***Asecodes*** |  |  |  |
| CO1, 1^st^ | F81 | Gamma | Dirichlet (1,1,1,1) |
| CO1, 2^nd^ | F81 | Gamma | Dirichlet (1,1,1,1) |
| CO1, 3^rd^ | HKY / GTR | Gamma | Dirichlet (1,1,1,1) |
| PGD | GTR | Equal | Dirichlet (1,1,1,1) |
| 28S | HKY | Equal | Dirichlet (1,1,1,1) |
| ITS | HKY | Equal | Fixed (equal) |

**Table SI-3**. The effect of the prior on the posterior estimate of the theta and tau parameters. The priors used are gamma distributions and the values given below, 0.1 and 0.01, are the means of the gamma distribution with alpha always being 1. The interval between the 2.50% and 97.50% includes 95% of the posterior probability distribution on the parameter. Upper part: the effect on tau posterior estimates. Note that the prior on tau only is a prior on the first split after the root. The posterior on other tau parameters (tau for the split between *Asecodes* on *G. tenella* and Asecodes on *G. pusilla/calmariensis* is given) are only affected in relation to the prior on the root split tau. With a prior of 0.1 on tau the data decreases the posterior estimate to 0.025-0.030, but with a prior of 0.01 on tau the data instead increases the posterior estimate to 0.020-0.026. This shows that the range between 0.1 and 0.01 definitively includes the posterior estimate. Lower part: the effect on theta posterior estimates. Note that both terminals and ancestors each have a separate theta parameter (seven in total for the four species model), and only two of these (theta for the root ancestor, and theta for the *Asecodes* species on *G. calmariensis/pusilla*) are shown in the table below. In contrast to tau, the prior on theta is a prior on all the theta parameters and the priors used were estimated to be close to the average over all seven theta parameters (not shown), as in BPP only a single theta prior can be specified. With a prior of 0.01 on theta the root ancestor theta parameter remains very close to 0.01 indicating this is a good prior. For the theta of the *Asecodes* species on *G. calmariensis/pusilla*, both a prior of 0.01 and 0.1 on theta are too low as in both cases the data increases the posterior estimate. All parameters in the table are estimated for the four species model and for the CO1 data (for the multilocus analysis a differential heredity multiplier is multiplied to all theta parameters for each locus to accommodate the difference between nuclear and mitochondrial effective population sizes).

|  |  |  |  | **Posterior tau(root)** | | | | **Posterior tau(cal_pus_ten)** | | | |
| --- | --- | --- | --- | --- | --- | --- | --- | --- | --- | --- | --- |
|  | Prior | theta | run | mean | S.D. | 2.5% | 97.5% | mean | S.D. | 2.5% | 97.5% |
| tau | 0.1 | 0.1 | 1 | 0.0238 | 0.0067 | 0.0130 | 0.0368 | 0.0036 | 0.0005 | 0.0025 | 0.0047 |
| (root) | 0.1 | 0.1 | 2 | 0.0257 | 0.0070 | 0.0108 | 0.0375 | 0.0036 | 0.0005 | 0.0025 | 0.0047 |
|  | 0.1 | 0.1 | 3 | 0.0254 | 0.0071 | 0.0118 | 0.0378 | 0.0036 | 0.0006 | 0.0024 | 0.0047 |
|  | 0.1 | 0.1 | 4 | 0.0268 | 0.0071 | 0.0116 | 0.0380 | 0.0036 | 0.0005 | 0.0025 | 0.0047 |
|  |  |  | **mean** | **0.0254** | **0.0070** | **0.0118** | **0.0375** | **0.0036** | **0.0006** | **0.0025** | **0.0047** |
| tau | 0.1 | 0.01 | 1 | 0.0299 | 0.0056 | 0.0172 | 0.0388 | 0.0035 | 0.0005 | 0.0025 | 0.0046 |
| (root) | 0.1 | 0.01 | 2 | 0.0307 | 0.0051 | 0.0187 | 0.0390 | 0.0034 | 0.0005 | 0.0024 | 0.0045 |
|  | 0.1 | 0.01 | 3 | 0.0302 | 0.0302 | 0.0178 | 0.0392 | 0.0035 | 0.0006 | 0.0024 | 0.0046 |
|  | 0.1 | 0.01 | 4 | 0.0297 | 0.0059 | 0.0169 | 0.0390 | 0.0035 | 0.0005 | 0.0024 | 0.0045 |
|  |  |  | **mean** | **0.0301** | **0.0117** | **0.0177** | **0.0390** | **0.0035** | **0.0005** | **0.0024** | **0.0045** |
| tau | 0.01 | 0.1 | 1 | 0.0226 | 0.0069 | 0.0101 | 0.0351 | 0.0035 | 0.0006 | 0.0024 | 0.0046 |
| (root) | 0.01 | 0.1 | 2 | 0.0177 | 0.0064 | 0.0080 | 0.0313 | 0.0035 | 0.0006 | 0.0022 | 0.0046 |
|  | 0.01 | 0.1 | 3 | 0.0206 | 0.0065 | 0.0105 | 0.0352 | 0.0036 | 0.0006 | 0.0024 | 0.0047 |
|  | 0.01 | 0.1 | 4 | 0.0195 | 0.0053 | 0.0103 | 0.0312 | 0.0036 | 0.0006 | 0.0024 | 0.0047 |
|  |  |  | **mean** | **0.0201** | **0.0063** | **0.0097** | **0.0332** | **0.0035** | **0.0006** | **0.0023** | **0.0046** |
| tau | 0.01 | 0.01 | 1 | 0.0259 | 0.0059 | 0.0145 | 0.0366 | 0.0035 | 0.0006 | 0.0024 | 0.0047 |
| (root) | 0.01 | 0.01 | 2 | 0.0283 | 0.0058 | 0.0156 | 0.0378 | 0.0035 | 0.0006 | 0.0024 | 0.0046 |
|  | 0.01 | 0.01 | 3 | 0.0272 | 0.0064 | 0.0132 | 0.0375 | 0.0034 | 0.0005 | 0.0024 | 0.0045 |
|  | 0.01 | 0.01 | 4 | 0.0246 | 0.0072 | 0.0103 | 0.0368 | 0.0034 | 0.0006 | 0.0022 | 0.0045 |
|  |  |  | **mean** | **0.0265** | **0.0063** | **0.0134** | **0.0372** | **0.0034** | **0.0006** | **0.0023** | **0.0046** |
|  |  |  |  |  |  |  |  |  |  |  |  |
|  |  |  |  | **Posterior theta(root)** | | | | **Posterior theta(cal_pus)** | | | |
|  | Prior | tau | run | mean | S.D. | 2.5% | 97.5% | mean | S.D. | 2.5% | 97.5% |
| theta | 0.1 | 0.1 | 1 | 0.0563 | 0.0611 | 0.0039 | 0.2240 | 0.2728 | 0.1292 | 0.0995 | 0.5978 |
|  | 0.1 | 0.1 | 2 | 0.0530 | 0.0578 | 0.0016 | 0.2116 | 0.2680 | 0.1249 | 0.0960 | 0.5760 |
|  | 0.1 | 0.1 | 3 | 0.0521 | 0.0571 | 0.0012 | 0.2132 | 0.2673 | 0.1278 | 0.0952 | 0.5793 |
|  | 0.1 | 0.1 | 4 | 0.0517 | 0.0613 | 0.0014 | 0.2249 | 0.2700 | 0.1323 | 0.0972 | 0.5955 |
|  |  |  | **mean** | **0.0533** | **0.0593** | **0.0020** | **0.2184** | **0.2695** | **0.1285** | **0.0970** | **0.5871** |
| theta | 0.1 | 0.01 | 1 | 0.0573 | 0.0590 | 0.0042 | 0.2197 | 0.2700 | 0.1281 | 0.1007 | 0.5878 |
|  | 0.1 | 0.01 | 2 | 0.0627 | 0.0594 | 0.0085 | 0.2238 | 0.2688 | 0.1257 | 0.0944 | 0.5815 |
|  | 0.1 | 0.01 | 3 | 0.0580 | 0.0570 | 0.0057 | 0.2182 | 0.2745 | 0.1334 | 0.0988 | 0.6051 |
|  | 0.1 | 0.01 | 4 | 0.0616 | 0.0614 | 0.0084 | 0.2320 | 0.2709 | 0.1266 | 0.0968 | 0.5787 |
|  |  |  | **mean** | **0.0599** | **0.0592** | **0.0067** | **0.2234** | **0.2710** | **0.1284** | **0.0977** | **0.5883** |
| theta | 0.01 | 0.1 | 1 | 0.0105 | 0.0096 | 0.0004 | 0.0348 | 0.0725 | 0.0204 | 0.0408 | 0.1201 |
|  | 0.01 | 0.1 | 2 | 0.0094 | 0.0089 | 0.0001 | 0.0323 | 0.0729 | 0.0202 | 0.0406 | 0.1198 |
|  | 0.01 | 0.1 | 3 | 0.0101 | 0.0092 | 0.0002 | 0.0347 | 0.0727 | 0.0200 | 0.0414 | 0.1183 |
|  | 0.01 | 0.1 | 4 | 0.0105 | 0.0097 | 0.0000 | 0.0354 | 0.0731 | 0.0199 | 0.0416 | 0.1198 |
|  |  |  | **mean** | **0.0101** | **0.0094** | **0.0002** | **0.0343** | **0.0728** | **0.0201** | **0.0411** | **0.1195** |
| theta | 0.01 | 0.01 | 1 | 0.0134 | 0.0100 | 0.0009 | 0.0385 | 0.0721 | 0.0199 | 0.0405 | 0.1180 |
|  | 0.01 | 0.01 | 2 | 0.0111 | 0.0096 | 0.0004 | 0.0353 | 0.0727 | 0.0199 | 0.0410 | 0.1190 |
|  | 0.01 | 0.01 | 3 | 0.0123 | 0.0101 | 0.0005 | 0.0382 | 0.0726 | 0.0199 | 0.0410 | 0.1180 |
|  | 0.01 | 0.01 | 4 | 0.0147 | 0.0108 | 0.0004 | 0.0418 | 0.0727 | 0.0201 | 0.0410 | 0.1193 |
|  |  |  | **mean** | **0.0129** | **0.0101** | **0.0006** | **0.0385** | **0.0725** | **0.0200** | **0.0409** | **0.1186** |

**Table SI-4**. Fragment lengths (bp), number of variable and parsimony informative sites and number of ingroup taxa for the *Galerucella* and *Asecodes* datasets.

|  | Fragment | # ingroup taxa | Sequence length, bp | Variable sites | Informative sites |
| --- | --- | --- | --- | --- | --- |
| ***Galerucella*** | CO1 | 51 | 1487 | 231 | 204 |
|  | 28S | 16 | 571 | 13 | 13 |
|  | ITS | 16 | 604 | 84 | 82 |
| ***Asecodes*** | CO1 | 107 | 1507 | 199 | 152 |
|  | PGD | 33 | 496 | 9 | 5 |
|  | 28S | 40 | 591 | 1 | 1 |
|  | ITS | 35 | 495 | 3 | 3 |

**Fig. SI-1.** Relationship between *Asecodes* specimen based on sequences of the nuclear genes PGD, 28S and ITS. Base pair changes of 28S and ITS are marked as black (28S) and grey (ITS) bars.
